# Time-Effective Simulation Methodology for Broadband Achromatic Metalens Using Deep Neural Networks

**DOI:** 10.3390/nano11081966

**Published:** 2021-07-30

**Authors:** Chun-Yuan Fan, Guo-Dung J. Su

**Affiliations:** Graduate Institute of Photonics and Optoelectronics, National Taiwan University, No. 1, Sec. 4, Roosevelt Rd, Taipei 10617, Taiwan; d05941014@ntu.edu.tw

**Keywords:** metasurface, local periodic approximation, broadband achromatic metalens, deep neural networks

## Abstract

Metasurface has demonstrated potential and novel optical properties in previous research. The prevailing method of designing a macroscale metasurface is based on the local periodic approximation. Such a method relies on the pre-calculated data library, including phase delay and transmittance of the nanostructure, which is rigorously calculated by the electromagnetic simulation. However, it is usually time-consuming to design a complex metasurface such as broadband achromatic metalens due the required huge data library. This paper combined different numbers of nanofins and used deep neural networks to train our data library, and the well-trained model predicted approximately ten times more data points, which show a higher transmission for designing a broadband achromatic metalens. The results showed that the focusing efficiency of designed metalens using the augmented library is up to 45%, which is higher than that using the original library over the visible spectrum. We demonstrated that the proposed method is time-effective and accurate enough to design complex electromagnetic problems.

## 1. Introduction

In nature, Abbe number nd−1nF−nC depicts an approximate measure of the material’s dispersion, where $n_{D}$, $n_{F}\;$, and $n_{c}$ are the refractive indices at the wavelengths of Fraunhofer at 589.3, 486.1, and 656.3 nm, respectively. To correct the chromatic aberration caused by the intrinsic property of materials, a doublet, which is composed of one crown and flint lenses, is a solution to the aberration in traditional optics. Previously, researchers have put effort into searching for new materials with different Abbe numbers and designing a complex system with several optical elements [1]. However, increasing the number of optical elements will cause the system to be bulkier and is inappropriate to be integrated with other sophisticated components. Subsequently, the next generation of optical components is diffractive optical elements (DOEs), significantly reducing the size and weight [2]. Rather than shape the refractive and reflective lens to be curved, DOEs modulate the phase of incident light by the principle of diffraction. Nevertheless, such optical components are suffered from crucial chromatic aberrations and thus restricted to several applications.

Metamaterials are three-dimensional artificial composite nanostructures that can manipulate electromagnetic waves and have versatile functionalities [3,4,5,6]. Metasurface, characterized by reducing the dimension of metamaterials, has attracted much attention due to integrating other optical components beyond the planar interfaces [7,8,9]. Rather than relying on gradual phase accumulation, each nanostructure causes an abrupt change in the phase of incident light [10]. When the light passes through the interface, such an abrupt phase change can arbitrarily deflect the light in any direction. A metalens is a kind of metasurface that manipulates the wavefront of light for focus [11,12]. Such a flat optical element enables it to overcome the spherical aberration that can occur in conventional lenses. Compared with traditional lenses, metalenses are flatter, thinner, and can correct the chromatic aberration with a single optical component. In previous research, broadband achromatic metalens has been researched and verified, including the theory of waveguide-mode or Pancharatnam–Berry phase-type [13,14]. The prevailing method of designing a metalens is based on the local periodic approximation (LPA) [15,16,17], the scattering in any small region is almost the same as the scattering from a periodic surface. Through the LPA approach, researchers can utilize the pre-calculated data library to design macroscale devices quickly. However, such an approach relies on the pre-calculated data library, including phase delay and transmittance of the nanostructure, which is calculated by the finite-difference time-domain (FDTD) solutions [18] or rigorous coupled-wave analysis (RCWA). Finding the most favorable nanostructure is time-consuming, especially for the multifunctional properties and broadband optical designs. How to effectively reduce the required time and computer resources based on the LPA method is an essential topic for the metasurface.

With the development of machine learning, some researchers have utilized neural networks to solve problems in different fields, including image recognition, speech recognition, and virtual data generation. The neural network allows computers to adaptively improve their performance with experience accumulated from the data observed. In the nanophononics field, deep neural network (DNN) and generative adversarial network (GAN) are applied to shape the nanostructure to fit the favorable parameters [19,20,21,22]. However, processing more complex nanostructure properties is a massive challenge due to the complex data structure causing nonlinear phase delay and transmission in the broadband wavelength. Moreover, abrupt change of the phase delay due to the standing wave will also cause the error. In the last few years, DNN has been used to acquire the relationship between the phase delay and the shape of three nanofin structures [23]. Another work utilized the GAN to learn the nanostructure mechanisms and compose new nanostructure shapes in the LPA method [24,25]. Such works demonstrated the advantages by combining the neural network in the electromagnetic fields. However, to improve the efficiency of the complex metasurface-based components such as broadband metalens [26,27], the phase delay and transmission for broadband wavelengths must also be considered.

In this paper, to establish a wide range of the group delay and high transmission in broadband wavelengths in our data library, we used the FDTD software to rigorously calculate the electromagnetic properties of different numbers of nanofins. The detail of FDTD settings and hardware were illustrated in the Supplementary Materials (Figure S1). Moreover, our previous studies utilized a similar method to extract the phase delay and transmission of nanostructures. [28,29]. Subsequently, the designed deep neural networks were trained by original data to predict the phase delay and the transmission for broadband wavelengths. Rather than the previous method to interpolate the data, the trained model can memorize the regular complex electromagnetic properties caused by the nanostructures. We used the well-trained model to predict the favorable nanostructures for designing a broadband achromatic for 400–650 nm in the visible wavelength. We demonstrated that the proposed method of combining deep neural networks is time-effective and reserves many computer resources to improve the focusing efficiency for complex electromagnetic problems. 

## 2. Materials and Methods

Broadband metasurface-based designs are widely utilized for optical properties, such as controlling light to focus or deflect through different wavelengths [30,31]. The fundamental method for designing broadband metalenses is to satisfy the phase delay, group delay, and group delay dispersion of the metalens at each spatial position to correct the spherical aberration and the chromatic aberration. For achromatic focusing at normal incidence, the relative phase *φ* provided by the metalens concerning the center is as follows [32]:

(1) $$\varphi \left(r,\omega \right)=-\frac{\omega }{c}\left(\sqrt{r^{2}+F^{2}}-F\right)$$

where *ω*, *r*, and *F* are angular frequency, the radial coordinate, and focal length. Since *φ(r,ω)* is spatial- and frequency-dependent, it implies that at a given r, different transverse wavevectors must be provided by the metalens to deflect the same angle at different wavelengths. Therefore, Equation (1) can be Taylor-expanded near a center frequency as follows [33]:

(2) $$\varphi_{metalens}\left(r,\omega \right)\;=\varphi \left(r,\omega_{d}\right)\;+{\frac{\partial \varphi \left(r,\omega \right)}{\partial \omega }|}_{\omega =\omega_{d}}\left(\omega -\omega_{d}\right)+{\frac{\partial^{2}\varphi \left(r,\omega \right)}{2\partial \omega^{2}}|}_{\omega =\omega_{d}}{\left(\omega -\omega_{d}\right)}^{2}+\ldots$$

**Figure 1:**
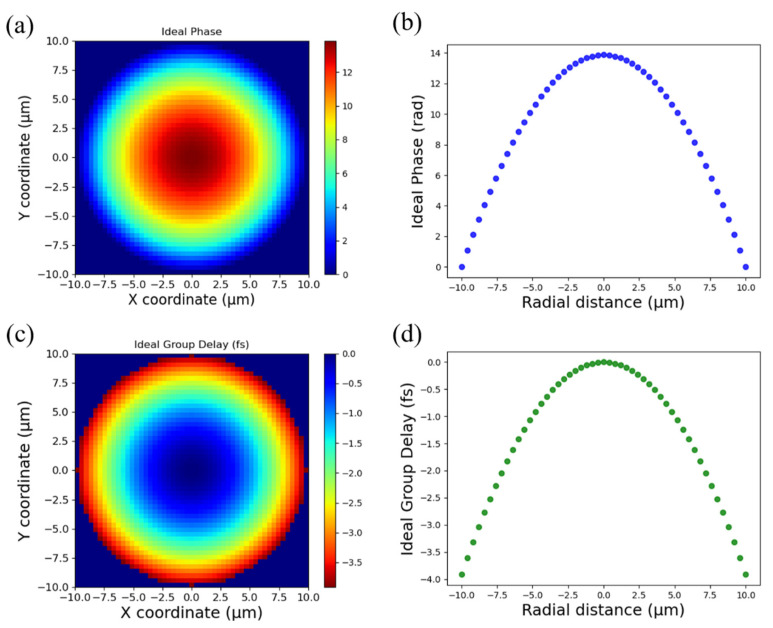
Design of the broadband achromatic metalens. (**a**) Ideal target phase for central wavelength at 532 nm. (**b**) Cross-section of ideal target phase for central wavelength. (**c**) Ideal group delay and (**d**) its cross-section. The aperture of the metalens is 20 μm, and the focal length is set to 42 μm.

Equation (2) indicates that to achieve achromatic focusing within a given bandwidth around $\omega_{d}$, and radial coordinate *r*, we need to satisfy the required relative phase and the group delay and higher-order derivative terms. To simulate the actual phase delay caused by the nanostructures, we designed a broadband achromatic metalens whose focal length is 42 μm and the aperture is 20 μm, and Figure 1 illustrates the ideal target phase for the 532 nm of center wavelength before subtraction of 2*π* and its required group delay.

We selected slot waveguide nanostructures composed of different numbers of nanofins to achieve high efficiency and a wide range of group delay properties. When a left-handed circularly polarized incident wave passed through the nanofin, the transmitted light can be illustrated by the Jones vector [34,35]:

(3) $$\frac{{\tilde{t}}_{L}+{\tilde{t}}_{S}}{2}\left[\begin{array}{c} 1\\ i \end{array}\right]+\frac{{\tilde{t}}_{L}-{\tilde{t}}_{S}}{2}\exp(i2\alpha )\left[\begin{array}{c} 1\\ -i \end{array}\right]$$

where ${\tilde{t}}_{L}$ and ${\tilde{t}}_{S}$ are the complex coefficients when the incident light is polarized along the long and short axis of the nanofin, and α is the rotation angle. We noticed that the second term in Equation (3) is cross-polarized and its normalized amplitude squared as the polarization conversion efficiency. The phase shift is illustrated by $\left({\tilde{t}}_{L}+{\tilde{t}}_{S}\right)\exp\left(i2\alpha \right)$.

Furthermore, previous researchers have assumed that the metasurface is locally periodic for each unit cell of aperiodic structures: Scattering in any small region is almost the same as scattering acquired from a periodic surface. Therefore, we rigorously calculated the phase delay and conversion efficiency using the commercial software Lumerical Inc. [36]. The materials used for our substrate and the nanostructures were sapphire and GaN, respectively. More detail of the refractive indices was shown in the Supplementary Materials (Tables S1 and S2) [37]. The unit cell and height of the nanostructures are set to 400 and 600 nm, respectively. The operating wavelength was from 400 to 650 nm. Previously, researchers took a significant amount of time and computer resources to establish the data library using the FDTD or RCWA method. However, it is difficult to find the most favorable nanostructure at each spatial position. The interpolation method is helpful for the simple nanostructure such as a single nanofin. However, such a method is not appropriate to the more complex structure and electromagnetic properties such as broadband designs. 

**Figure 2:**
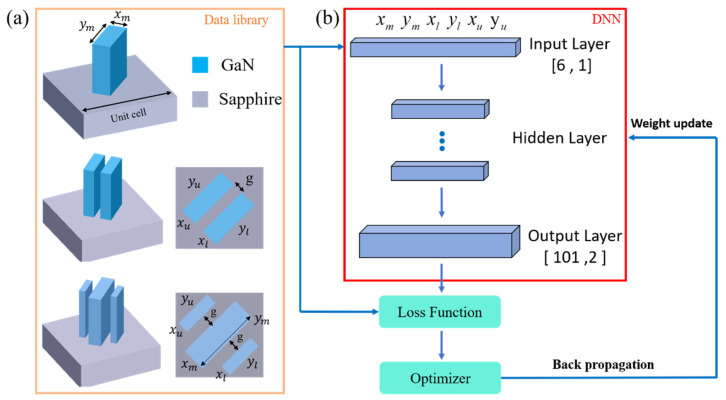
Proposed methodology for the broadband achromatic metalens. (**a**) The original data library includes different numbers of nanofins calculated by the FDTD method. (**b**) The architecture of deep neural network where the output layer is the phase delay and the conversion efficiency. There are 101 steps at wavelengths from 400 to 650 nm. The gap is 60 nm.

Therefore, in this paper, we proposed a time-effective methodology for broadband achromatic metalens using deep neural networks, and the operational flow was shown in Figure 2a,b. First, we used the FDTD method to establish about three hundred data for each different number of nanofins and calculated the phase delay and conversion efficiency in broadband wavelengths in 101 steps. Second, we utilized DNN to realize the inverse design of our different numbers of nanofins. The input layer included six parameters which illustrated the long and short axis for the middle, lower, and upper nanofin. For example, the long and short axis for the lower and upper nanostructure will be zero for a single nanofin in the input layer. Second, the long and short axis for the middle nanostructure will be zero for double nanofins. The output layer included the phase delay and conversion efficiency at wavelengths from 400 to 650 nm in 101 steps. After several epochs, the weights for each hidden layer were continuously updated until the loss function was small enough. The well-trained DNN will be utilized to predict the phase delay and conversion efficiency in broadband according to the given input layer. The original data library could be further extended rapidly, combining with the FDTD method and the deep neural network. Compared to the previous method to establish a data library, the proposed methodology has significantly reduced the time and computer resources to establish a vast data library covering a wide range of group delays and conversion efficiencies.

## 3. Results and Discussion

**Figure 3:**
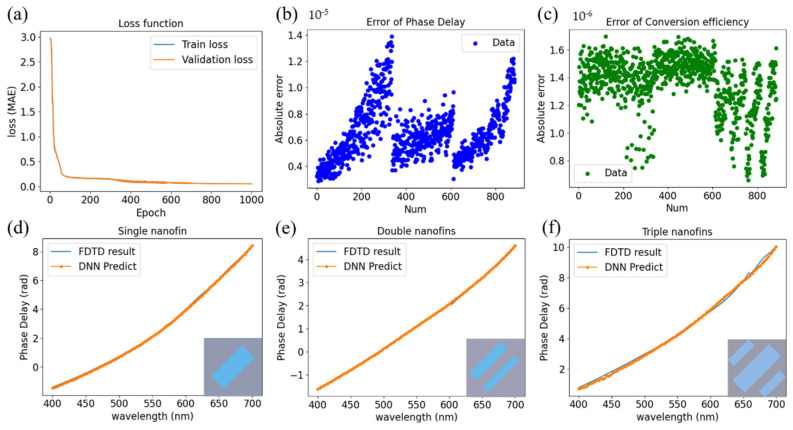
The detail and the results of deep neural network (**a**) loss function measure using the mean absolute error (MAE) according to the train and validation loss, respectively. (**b**) Error of phase delay and (**c**) error of conversion efficiency. (**d**–**f**) The comparison of FDTD result and the DNN predict. The parameters for the short and long axis ($x_{m}$,$\;y_{m}$,$\;x_{u},y_{u},x_{l},y_{l}$) in (**d**–**f**) are (120, 300, 0, 0, 0, 0), (0, 0, 70, 300, 50, 300), and (110, 350, 55, 200, 65, 210), respectively. The unit is in nanometer.

To verify the validity of the proposed method, we showed the detail and the results of the proposed DNN in this section. The total number of input data composed of three different numbers of nanofins were about 1000 groups and rigorously calculated by the FDTD method. We modified Pytorch, an open-source machine learning framework, to establish our deep neural network [38,39]. The input data was normalized and randomly separated into train and validation datasets. The hidden layer was made of seven layers, and each layer was calculated by the rectified linear unit (ReLU), which was beneficial to train and achieve better performance. Each layer has several neurons, and the hyperparameters such as learning rate and batch size are set to $5\times 10^{-4}$ and complete batch learning. After every epoch, the weight and the bias will be optimized by the adaptive moment estimation (Adam) to decrease the loss value. Figure 3a shows the loss function versus epoch which created a criterion that measures the mean absolute error (MAE) according to the train and validation loss. Figure 3b,c shows the absolute error of the phase delay and conversion efficiency between the results calculated by the FDTD and predicted by the DNN model. 

Moreover, to further demonstrate the accuracy of the designed model, we also randomly selected three different numbers of nanofins that were nonexistent in the original library to the phase delay calculated by FDTD and DNN through the wavelengths from 400 to 700 nm in Figure 3d–f. It is noticed that the phase delay of the result was not with 2π subtraction due to the convenience of calculating the group delay. Figure 3d–f shows that the phase delay is not entirely linear, which is caused by the intrinsic property of materials. Therefore, we select the range from 400 to 650 nm to calculate the rate of change of the total phase shift.

**Figure 4:**
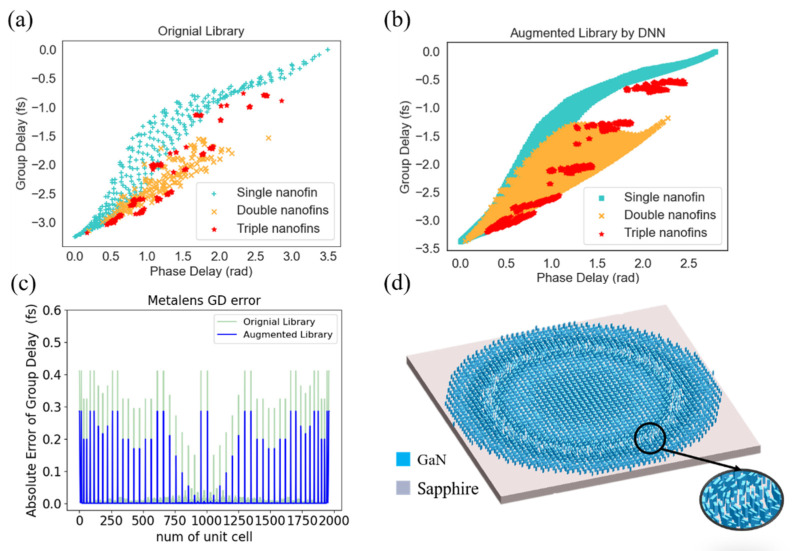
The data and absolute error of original and augmented library and the designed broadband achromatic metalens schematic. (**a**) The FDTD simulation calculated the original data library (one thousand points). (**b**) The augmented data library (ten thousand points) predicted by the DNN model. (**c**) A comparison between the group delay error selected from the original and augmented library. The horizontal axis is the unit cell on the metalens. (**d**) Schematic of the broadband achromatic metalens constructed by the augmented library. The materials of substrate and nanostructures are sapphire and GaN, respectively.

After the linear fitting, we calculated the group delay and established a new augmented data library predicted by the DNN model. Owing to the broadband wavelengths, complex electromagnetic properties, and high mesh accuracy, each point in Figure 4a took about 10–20 min. Therefore, establishing a vast data library is very time-consuming. Figure 4a,b showed the original data library (one thousand points) calculated by FDTD simulation and the augmented data library (ten thousand points) predicted by the DNN model. From the results of the figure, the points in the augmented library were much denser than the original library and thus were time-effective and reserved many computer resources. To achieve broadband achromatic metalens, nanostructures at each position should simultaneously satisfy phase delay, group delay, and group delay dispersion [26,27]. Therefore, we first used the root mean square method to find the most approximate group delay at each spatial position from the data library. Subsequently, each nanostructure was rotated to satisfy the required phase delay followed by the Pancharatnam-Berry phase, which depicts the phase shift generated by the rotation and can be shown as φ(x,y)=2θ(x,y). 

Furthermore, the group delay dispersion was close to zero since both the phase delay and group delay were accurately matched. Figure 4c shows the absolute group delay error of the original and augmented library compared to the ideal group delay, which was calculated in Figure 1. The horizontal axis is the unit cell on the metalens. The absolute error selected from the augmented library is lower than the original library. More importantly, the augmented data library provided more possibilities (ten times more data points) to select the nanostructures that accurately match the target group delay and have high conversion efficiency (reducing the absolute error group delay by 25~90%). Figure 4d illustrated the schematic of designed broadband achromatic metalens whose focal length is 42 μm and NA is about 0.23, and the operating wavelength is from 400 to 650 nm. 

**Figure 5:**
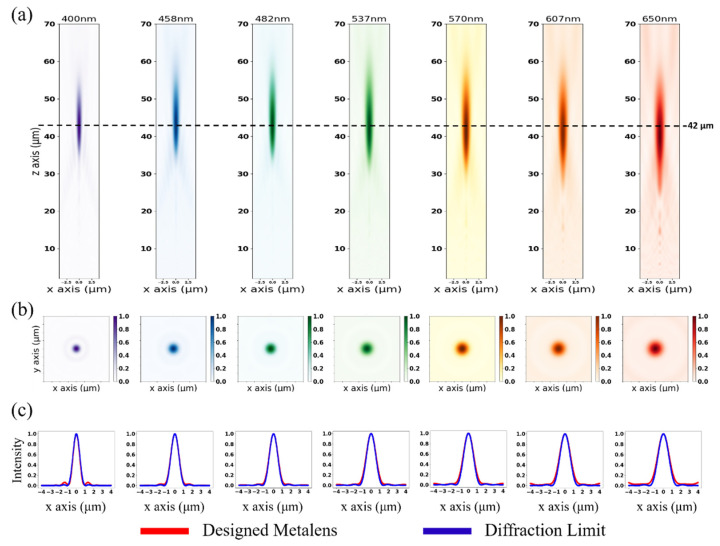
Far-field plots of broadband achromatic metalens for x-z, x-y, and cross-section along the *X*-axis. (**a**) Far-field on the x-z plane. (**b**) Far-field on the x-y plane. (**c**) Cross-section along the *X*-axis at the focus. The designed focus and NA are 42 μm and 0.23, and the operating wavelengths are from 400 to 650 nm.

To verify the proposed design, we used the FDTD method to analyze electromagnetic propagation by employing near-to-far-field transformation [40] to calculate the image quality at different wavelengths from 400 to 650 nm. Figure 5a,b presents the normalized intensity distribution results in the x-z, x-y plane, and the foci are approximate to the designed focal length and NA, which are 42 μm and 0.23, respectively. Figure 5c illustrates the cross-section along the *X*-axis at the far-field in the x-y plane. To analyze image quality at multiple wavelengths, we used the normalized intensity of the airy disk adhered to the Fraunhofer diffraction pattern of a circular aperture, which is expressed as:

(4) $$I\left(\theta \right)=I_{0}{\left(\frac{2J_{1}\left(ka\sin\theta \right)}{ka\sin\theta }\right)}^{2}$$

where $I_{0}$ is the maximum intensity distribution of the pattern at the Airy disk center, $J_{1}$ is the Bessel function of the first kind of order one, *k* is the wavenumber, a is the radius of the aperture, and *θ* is the observation angle. We compared cross-sections between the designed metalens and diffraction limit at seven different wavelengths. It is worth mentioning that the phase delay caused by the GaN is not entirely linear from 400 to 700 nm, and thus caused a slight mismatch at 650 nm wavelength. Nonetheless, the results show that the full width at half maximum (FWHM) of designed metalens almost approaches the diffraction limit at different wavelengths. 

**Figure 6:**
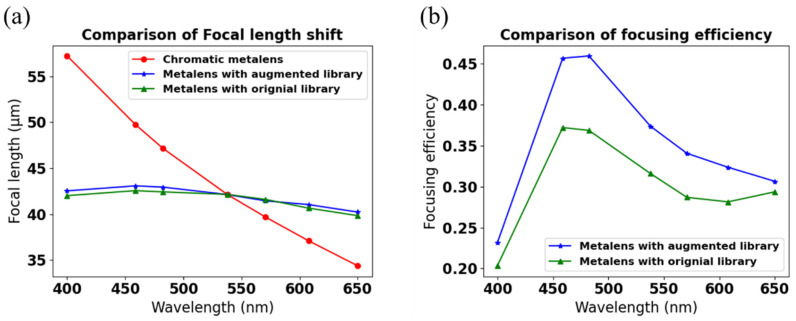
Comparison of the focal length shift and focusing efficiency at operating wavelengths. (**a**) Focal length shift; (**b**) focusing efficiency.

Furthermore, we demonstrated the advantages of the designed broadband achromatic metalens constructed by the augmented data library. We performed the error analysis of the phase shift and calculated focusing efficiencies at different wavelengths. The focusing efficiency is the fraction of incident light that passes through the metalens divided by the area of a circular iris on the focal plane with thrice the radius of the FWHM spot size [41,42]. Figure 6a shows the comparison of the focal length shift in three different cases. The result shows that the focal shift is approximately ±5% in metalens with the augmented and library, simultaneously satisfying the phase delay and group delay. On the other hand, the focal shift of chromatic metalens is up to about ±30%. Figure 6b shows the comparison of focusing efficiency between the metalens with the augmented and original library. The augmented library’s focusing efficiency is higher than the original library since the augmented library has massive data to make us find the high conversion efficiency with accurate group delay. The focusing efficiency can be as high as 45% using the augmented data library from 450–500 nm, as shown in Figure 6b. 

**Figure 7:**
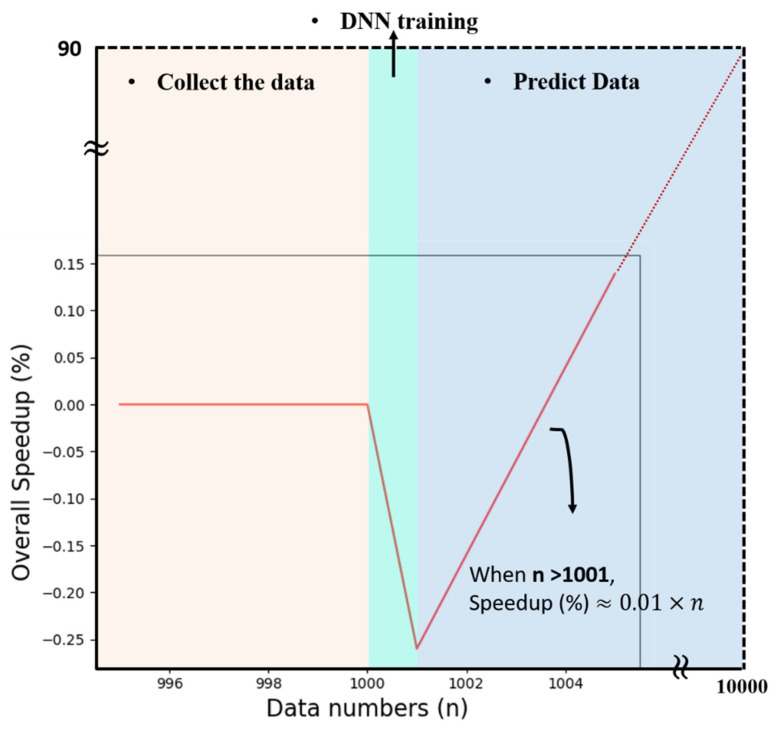
Overall speedup for the proposed method combining the FDTD method and DNN model. The horizontal axis is data numbers. The vertical axis is overall speedup.

Establishing a massive data library with only the FDTD method will be time-consuming and cost many computer resources. The proposed method can be used to increase the data numbers in the library rapidly and thus find the most favorable structures. However, such a method required original data, which is large enough to learn the electromagnetic properties. In this paper, we used the FDTD method to establish about one thousand data with high accuracy, and each data takes an average of 15 min. Subsequently, we utilized the DNN model to predict about ten thousand data, and such work can be finished in a half-hour, including training the model. If we simulate ten thousand data with only the FDTD method, it is unrealistic even though the results are more accurate than the proposed method. Figure 7 shows the overall speedup for the original method and the proposed method. The speedup is defined as the ratio of the reduced time and the original time. The positive numbers mean the needed time is reduced. We could see negative numbers during the DNN training period, which are marginal. When the numbers of data are more than 1001, the overall speedup is proportional to the data numbers. The overall speedup is slightly lower at the 1001st data due to the training time in DNN, but increases significantly when the data numbers increase. For example, when we increase the data numbers from one thousand to ten thousand points, we could save up to 90% of the time needed for the original method. We simulated the far-field distributions with ultrahigh accuracy, and it shows that the augmented data is accurate enough to design a broadband achromatic metalens. Such a design with the neural network allows it to play a significant role in the electromagnetic fields.

## 4. Conclusions

This paper proposes a time-effective methodology for broadband achromatic metalens by modifying deep neural networks. Compared to the previous researches, we combined different numbers of nanofins and used deep neural networks to train our data library, and the well-trained model predicted the new data, which has a higher transmission for designing a broadband achromatic metalens. The results revealed that the FWHM of the designed metalens using the augmented library approaches the diffraction limit at broadband wavelengths from 400 to 650 nm. Moreover, the focusing efficiencies at all wavelengths increased due to the more alternative nanostructures in the augmented data library established by the DNN model. The focusing efficiency can be as high as 45% using the augmented data library. Such method combining the FDTD simulation and deep neural network is time-consuming and reserves many resources to design complex electromagnetic problems. 

## Data Availability

The study did not report any data.

## Supplementary Materials

The following are available online at www.mdpi.com/article/10.3390/nano11081966/s1, Figure S1: Schematic of the broadband achromatic metalens. Table S1: The refractive index of GaN in the database. Table S2: The refractive index of Sapphire in the database.
