# Supplementary material for: Time-Effective Simulation Methodology for Broadband Achromatic Metalens Using Deep Neural Networks
Source: Nanomaterials (Basel). 2021 Jul 30;11(8):1966. doi: 10.3390/nano11081966 (PMC8398648; doi:10.3390/nano11081966)
Supplement: Supplementary file 1 [file nanomaterials-11-01966-s001.zip › nanomaterials-1286631-supplementary.pdf]

nanomaterials

Supplementary Materials

Time-Effective Simulation Methodology for Broadband Achromatic Metalens Using Deep Neural Networks

Chun-Yuan Fan and Guo-Dung J. Su *

Graduate Institute of Photonics and Optoelectronics, National Taiwan University, No.1, Sec.4, Roosevelt Rd, Taipei 10617, Taiwan; d05941014@ntu.edu.tw
* Correspondence: gdjsu@ntu.edu.tw

Methods

Simulations were executed using 3D finite-difference time-domain method (FDTD solutions from Lumerical Inc.) [1]. Different numbers of nanofins were simulated under circularly polarized illumination according to different lengths, widths. Periodic and perfectly matched layer boundary conditions were utilized along with the transverse and longitudinal directions according to light propagation. The thickness of the substrate was the same as the simulation, which is equal to the unit cell (400 nm). The source was considered as a plane wave in the substrate, and the distance between the source and the bottom of the nanostructure was set to 0.5 μm. The mesh accuracy was fixed to 4 in the mesh settings corresponding to 18 mesh points per wavelength. The time step was set to about 0.032 fs. The schematic of the FDTD setting was shown in Fig. S1. Furthermore, we used metamaterial S parameter extraction, in which was provided by Lumerical software. to extract the phase delay and transmission of the nanostructure. After the linear fitting, we calculated the group delay and established the data library used in the paper.

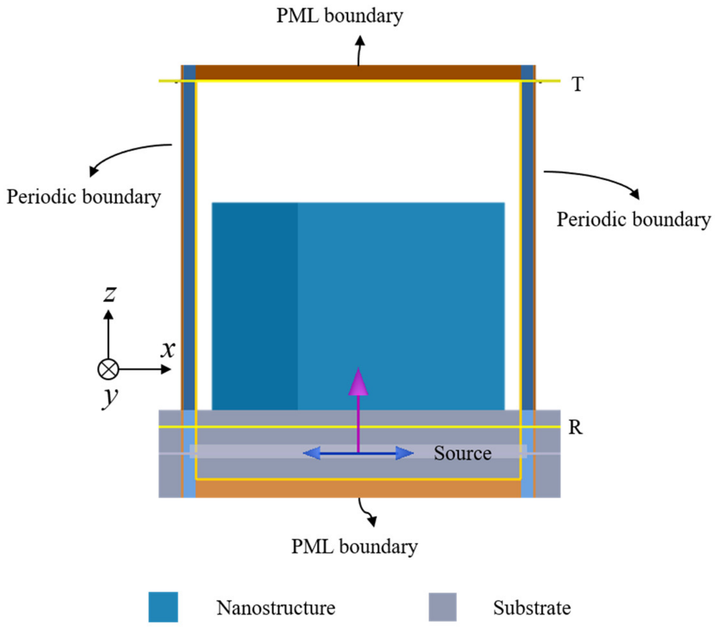

Figure S1. Schematic of the broadband achromatic metalens.

Hardware

Processors: Intel(R) Xeon (R) Silver 4110 CPU
Display adapters: NVIDIA GeForce RTX 2080 Ti
RAM: 128 GB

Materials

We import the refractive indices of GaN and Sapphire from RefractiveIndex.INFO [2] to Lumerical simulation software. The RefractiveIndex.INFO is an open database, and the refractive index used in our paper was shown in Table 1 and Table 2.

Table S1. The refractive index of GaN in the database.

Wavelength (μm).	Refractive Index of GaN
0.4002	2.5612456085888
0.4139	2.5361462810057
0.4280	2.5144550692436
0.4426	2.4954494513073
0.4577	2.478711043587
0.4733	2.4639009199004
0.4894	2.4507417483261
0.5061	2.4389387363249
0.5233	2.4283841176326
0.5412	2.4188141907328
0.5596	2.410215638829
0.5787	2.4023892693832
0.5984	2.3952923706351
0.6188	2.3888143189414
0.6399	2.3828947668339
0.6618	2.3774559658183
0.6843	2.3724983348334
0.7076	2.3679330099592

Table S2. The refractive index of Sapphire in the database.

Wavelength (μm)	Refractive Index of Sapphire
0.4060	1.7855131978094
0.4193	1.7834376078764
0.4330	1.7815032886078
0.4472	1.7796876970942
0.4618	1.7779954669163
0.4770	1.7763969806669
0.4926	1.7749069668851
0.5087	1.7735082113872
0.5253	1.7721946418157
0.5425	1.7709534510436
0.5602	1.7697870034397
0.5786	1.768678092594
0.5975	1.7676347594555
0.6170	1.7666467187031
0.6372	1.7657055418929
0.6581	1.7648083776856
0.6796	1.7639560666816
0.7018	1.7631412029905

References

1. Lumerical Solutions, Inc. Available online: http://www.lumerical.com/tcad-products/fdtd/.
2. Polyanskiy, M.N. Refractive Index Database. Available online: https://refractiveindex.info (accessed on 19 July 2021).
